# Colorectal Cancer Care Delivery in Rural and Socioeconomically Deprived Areas of Maine

**DOI:** 10.46804/2641-2225.1258

**Published:** 2026-05-28

**Authors:** Christina A. Kapala, Kimberly Mooney, Sydney Bebus, Lise M. Cloutier, John L. Daggett, Benjamin R. Felix, Renee M. Fay-Leblanc, Adriana E. Nadeau, Bridget K. Rauscher, Kathryn Rensenbrink, Debra A. Rothenberg, Kevin D. Stein, Kathleen M. Fairfield

**Affiliations:** a Department of Internal Medicine, MaineHealth MidCoast Medical Center, Bath, Maine; b MaineHealth Institute for Research, Scarborough, Maine; c Harrington Family Health Center, Harrington, Maine; d Department of Internal Medicine, Central Maine Healthcare, Lewiston, Maine; e Department of Internal Medicine, MaineHealth Maine Medical Center, Portland, Maine; f Greater Portland Health, Portland, Maine; g Western Maine Health Internal Medicine, Norway, Maine; h Portland Community Free Clinic, City of Portland, Portland, Maine; i Department of Internal Medicine, Northern Light Health, Ellsworth, Maine; j Department of Family Medicine, MaineHealth Maine Medical Center, Portland, Maine

**Keywords:** Medicaid, Access to care, Healthcare disparities, Rural health

## 1. Introduction

People living in rural regions and those experiencing socioeconomic deprivation are known to have poorer cancer outcomes.^1–4^ In prior work, we found that a later stage at diagnosis for colorectal cancer (CRC) was associated with living in rural areas or having Medicaid insurance, but there were no differences according to local Area Deprivation Index (ADI).^5^ These findings suggested poor access to screening or symptom evaluation. Cancer treatment is demanding, and people in rural and low-income areas may have difficulty with transportation and other support needed to engage in cancer care.^6^ Our goal was to characterize relationships between rurality and area socioeconomic deprivation and treatment for CRC in Maine.^7^ A better understanding of the drivers of cancer care variation will help inform health policy and health system changes to improve access to care.

## 2. Methods

### 2.1. Cancer registry data

We obtained deidentified data from the Maine Cancer Registry for all incident CRC cases in adults (21 years and older) from 2017 to 2021. The stage at diagnosis was defined by the SEER Summary Stage Manual (https://seer.cancer.gov/tools/staging/index.html). We collapsed the stages into groups—local, regional, and distant spread—and excluded cases with in-situ stage for this analysis (n = 89). For analyses of treatment differences, we separated colon cancers (n = 1988) from rectal (n = 883) and anal (n = 214) cancers.

### 2.2. Rurality and area deprivation index

Rurality was determined by mapping 5-digit ZIP codes to the 2010 Rural-Urban Commuting Area (RUCA) Codes (https://depts.washington.edu/uwruca/ruca-uses.php).^8^ We grouped RUCA codes into tiers: urban, large rural, small rural, and isolated rural. To examine the impact of poverty in addition to rurality, we used the state-level ADI that was ascertained using a crosswalk from the University of Wisconsin Neighborhood Atlas (https://www.neighborhoodatlas.medicine.wisc.edu/). ADI is a composite measure of neighborhood socioeconomic disadvantage based on factors such as income, education, employment, and housing quality ranked from 1 (least disadvantaged) to 10 (most disadvantaged) at the state level. We collapsed groups into quintiles to ensure adequate statistical power without losing the ability to discriminate between groups. We excluded people for whom we could not assign RUCA (n = 4) and ADI (n = 45) because this factor was our primary interest for this study. After the exclusions, 3085 cases were included in the analysis.

### 2.3. Statistical analysis

We used descriptive statistics (chi-square and t tests) to describe the overall population of CRC cases in the registry. Using R version 4.2.1, separate logistic regression models were constructed with treatment (surgery, chemotherapy, or radiation) as the dependent variable, with separate models for colon and rectal/anal cancers. Independent variables included age, sex, race, insurance type, rurality, ADI, stage, and year of diagnosis. The MaineHealth Institutional Review Board approved this study.

## 3. Results

Table 1 shows the demographics of the 3085 CRC cases. Most cases occurred in older adults with a median (SD) age of 67 (14) years. Cases were split evenly by sex; 98% (3016 of 3085) were White and the remaining 2% (69 of 3085) were another or unknown race. Medicare (43% [1335 of 3085]) or commercial insurance (31% [969 of 3085]) were most common. Also, 34% (1063 of 3085) of people resided in small or isolated rural areas, and 43% (1323 of 3085) resided in areas in the highest 2 quintiles of ADI. Regional or distant spread at diagnosis was present among 60% (1902 of 3085) of cases. Cases were diagnosed evenly across calendar years.

**Fig. 1.**
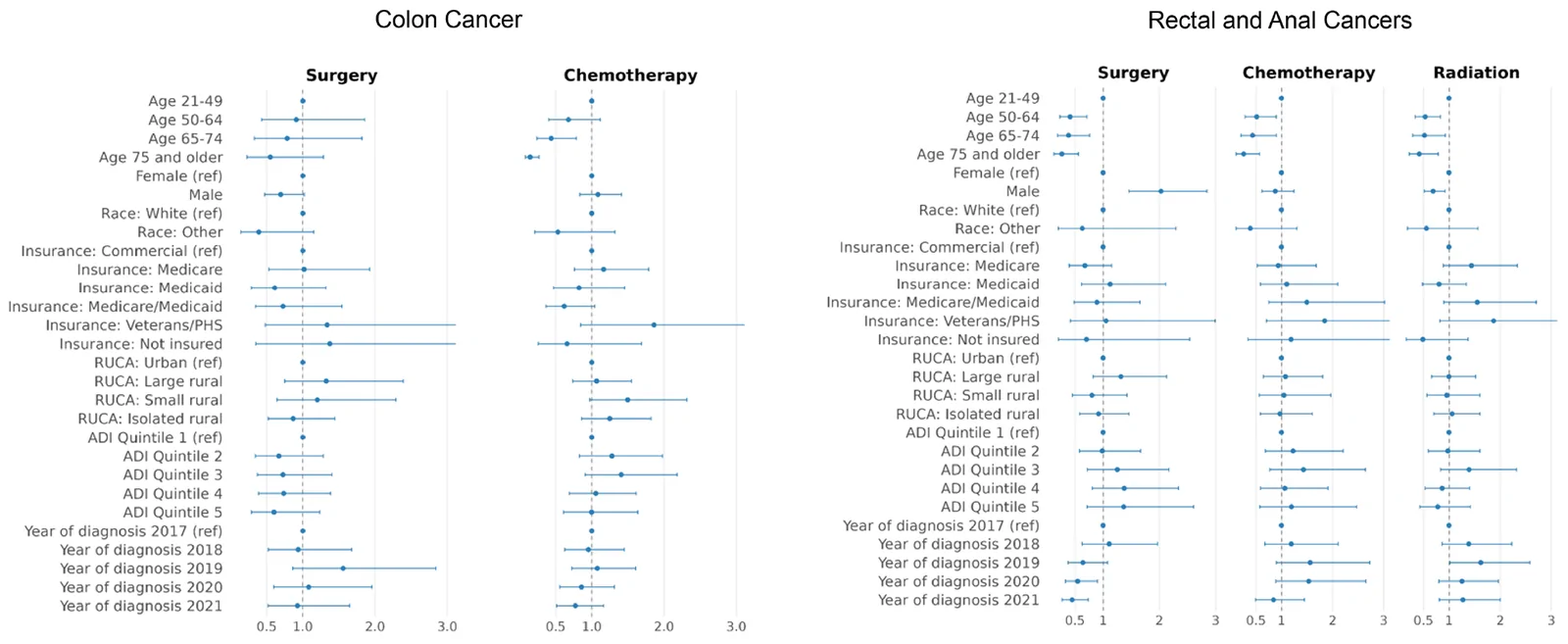
Forest Plots of Adjusted Odds Ratios of Treatment with Surgery and Chemotherapy according to Rurality and ADI from Multivariable Models by Cancer. ADI, Area Deprivation Index, and Insurance Status.

No differences in treatment according to rurality and ADI were observed in bivariate analyses (data not shown). In multivariable logistic regression models (Fig. 1) with treatment (surgery, chemotherapy, or radiation) as the dependent variable, we observed no differences in the odds of receiving treatment according to rurality or ADI (see Appendixes for odds ratios and confidence intervals). Because so few people with colon cancer received radiation (n = 43), we do not show the radiation findings for colon cancer (Fig. 1, Appendix A). We observed lower odds of receiving chemotherapy among older patients with colon cancer, lower odds of all treatment types among older patients with rectal/anal cancers, and lower odds of radiation among men with rectal/anal cancer (Appendix B). We also observed lower odds of receiving surgery for people with rectal/anal cancer diagnosed in 2019 through 2021.

## 4. Discussion

We found no difference in the treatment for colon, rectal, and anal cancers in rural dwellers or people living in areas with high area poverty. Older people (75 years and older) had lower odds of receiving treatment compared with people ages 50 years and younger, which may be clinically appropriate. The COVID-19 pandemic resulted in decreased rates of cancer diagnoses nationally in 2020 and 2021,^7^ although we did not observe this trend. Changes in treatment guidelines for rectal and anal cancers may explain the lower odds of receiving surgery and higher radiation use for these cancers during this period. Our findings contradict several studies summarized in a recent review that described CRC treatment disparities in rural populations^9^ and other studies showing rural-urban differences.^11,12^ Because the population in Maine is predominantly White, we are unable to adequately examine racial differences in treatment or the intersectionality of race and small area deprivation together, as others have done.^10^

Our study is limited to a single state cancer registry. We do not have information on comorbidities to determine whether treatment was medically indicated, particularly to understand the treatment variation we observed among older patients. We also did not have access to survival data. The study may not be generalizable across the United States or between states, in part because the demographics of Maine are relatively homogeneous with regard to race. Specifically, it would be difficult to generalize our findings to more urban areas of the United States, areas with higher Medicaid populations, or other rural areas with higher racial, ethnic, and age diversity. In addition, we had a limited numbers of cases, particularly rectal and anal cancers, to explore differences in treatment.

In summary, we found no consistent evidence of variation in care for colon, rectal, and anal cancers according to rurality or area level poverty in Maine. Future efforts to understand colorectal care quality for people living in rural and low-income areas will require a more nuanced analysis of barriers to care and clinical outcomes data, particularly in older adults.

## Figures and Tables

**Table 1.** Characteristics of Patients with Incident Colorectal Cancer, Maine Cancer Registry, 2017–2021 (N = 3085).

Characteristic	Overall n = 3085	Colon n = 1988	Rectal/Anal n = 1097
Age at diagnosis, median (SD), y	67 (14)	69 (14)	63 (13)
Age group, No. (%), y			
21-49	338 (11)	177 (8.9)	161 (15)
50-64	993 (32)	548 (28)	445 (41)
65-74	873 (28)	576 (29)	297 (27)
75 and older	881 (29)	687 (35)	194 (18)
Sex, No. (%)			
Female	1552 (50)	1036 (52)	516 (47)
Male	1533 (50)	952 (48)	581 (53)
Race, No. (%)			
White	3016 (98)	1940 (98)	1076 (98)
Other/Unknown	69 (2.2)	48 (2.4)	21 (1.9)
Insurance, No. (%)			
Commercial	969 (31)	563 (28)	406 (37)
Medicare	1335 (43)	938 (47)	397 (36)
Medicaid	233 (7.6)	127 (6.4)	106 (9.7)
Medicare/Medicaid	332 (11)	218 (11)	114 (10)
Veterans/public health service	110 (3.6)	69 (3.5)	41 (3.7)
Not insured	61 (2.0)	40 (2.0)	21 (1.9)
Unknown	45 (1.5)	33 (1.7)	12 (1.1)
RUCA category, No. (%)			
Urban	1469 (48)	946 (48)	523 (48)
Large rural	553 (18)	351 (18)	202 (18)
Small rural	442 (14)	298 (15)	144 (13)
Isolated rural	621 (20)	393 (20)	228 (21)
ADI quintile, No. (%)			
ADI Q1	466 (15)	313 (16)	153 (14)
ADI Q2	613 (20)	385 (19)	228 (21)
ADI Q3	683 (22)	436 (22)	247 (23)
ADI Q4	766 (25)	483 (24)	283 (26)
ADI Q5	557 (18)	371 (19)	186 (17)
Stage at diagnosis, No. (%)			
Localized	1183 (38)	755 (38)	428 (39)
Regional	1313 (43)	845 (43)	468 (43)
Distant	589 (19)	388 (20)	201 (18)
Year of diagnosis, No. (%)			
2017	612 (20)	400 (20)	212 (19)
2018	589 (19)	393 (20)	196 (18)
2019	658 (21)	443 (22)	215 (20)
2020	591 (19)	346 (17)	245 (22)
2021	635 (21)	406 (20)	229 (21)
Surgery, No. (%)	2484 (84)	1762 (90)	722 (71)
NA	120	40	80
Chemotherapy, No. (%)	1370 (50)	678 (40)	692 (68)
Missing	355	275	80
Radiation, No. (%)	644 (22)	43 (2.2)	601 (58)
Missing	100	44	56

Abbreviations: ADI, Area Deprivation Index; RUCA, Rural-Urban Commuting Area; NA, Patients for whom treatment was too risky, died, or refused treatment were excluded in each treatment specific category.

## Appendix

**A.** Logistic Regression Results for Treatment Outcomes: Colon Cancer

	Surgery			Chemotherapy		
Variable	Odds Ratio	95% CI	P-value	Odds Ratio	95% CI	P-value
Age Group			0.3			<**0.001**
Age 21–49	—	—		—	—	
Age 50–64	0.91	0.43, 1.86		0.68	0.41, 1.12	
Age 65–74	0.78	0.33, 1.82		0.44	0.24, 0.79	
Age 75 and older	0.55	0.23, 1.29		0.15	0.08, 0.27	
Sex			0.062			0.5
Female	—	—		—	—	
Male	0.70	0.47, 1.02		1.09	0.83, 1.41	
Race			0.087			0.2
White	—	—		—	—	
Other/Unknown	0.39	0.14, 1.15		0.53	0.21, 1.32	
Insurance			0.7			0.059
Commercial	—	—		—	—	
Medicare	1.02	0.53, 1.92		1.16	0.76, 1.79	
Medicaid	0.61	0.29, 1.32		0.82	0.47, 1.45	
Medicare/Medicaid	0.73	0.35, 1.54		0.62	0.37, 1.04	
Veterans/public health service	1.34	0.48, 4.03		1.86	0.84, 4.20	
Not insured	1.37	0.35, 6.99		0.66	0.26, 1.69	
Unknown	0.60	0.14, 3.20		0.72	0.23, 2.17	
RUCA Category			0.5			0.3
Urban	—	—		—	—	
Large rural	1.32	0.75, 2.39		1.07	0.73, 1.55	
Small rural	1.20	0.64, 2.29		1.50	0.97, 2.32	
Isolated rural	0.87	0.52, 1.45		1.25	0.86, 1.82	
ADI Quintile			0.7			0.4
ADI_Q1	—	—		—	—	
ADI_Q2	0.67	0.34, 1.28		1.28	0.83, 1.98	
ADI_Q3	0.73	0.37, 1.40		1.41	0.91, 2.18	
ADI_Q4	0.74	0.39, 1.38		1.06	0.69, 1.61	
ADI_Q5	0.60	0.29, 1.24		1.00	0.61, 1.64	
Year of diagnosis			0.4			0.5
2017	—	—		—	—	
2018	0.94	0.52, 1.68		0.95	0.63, 1.45	
2019	1.56	0.86, 2.84		1.08	0.72, 1.61	
2020	1.08	0.60, 1.96		0.86	0.56, 1.31	
2021	0.93	0.52, 1.65		0.77	0.51, 1.16	
Stage at diagnosis			**<0.001**			**<0.001**
Localized	—	—		—	—	
Regional	1.26	0.59, 2.71		33.3	22.9, 50.0	
Distant	0.02	0.01, 0.04		83.2	53.6, 133	

Abbreviations: CI = Confidence Interval, OR = Odds Ratio.

**B.** Logistic Regression Results for Treatment Outcomes: Rectal/Anal Cancer

Variable	Surgery			Chemotherapy			Radiation		
	Odds Ratio	95% CI	P-value	Odds Ratio	95% CI	P-value	Odds Ratio	95% CI	P-value
Age Group			**0.002**			**0.008**			**0.022**
Age 21–49	—	—		—	—		—	—	
Age 50–64	0.41	0.24, 0.71		0.52	0.29, 0.90		0.53	0.34, 0.83	
Age 65–74	0.39	0.19, 0.76		0.44	0.21, 0.90		0.52	0.29, 0.93	
Age 75 and older	0.27	0.13, 0.56		0.26	0.12, 0.57		0.42	0.22, 0.79	
Sex			**<0.001**			0.5			**0.012**
Female	—	—		—	—		—	—	
Male	2.03	1.46, 2.84		0.88	0.62, 1.25		0.69	0.51, 0.92	
Race			0.5			0.13			0.3
White	—	—		—	—		—	—	
Other/Unknown	0.63	0.21, 2.29		0.39	0.12, 1.30		0.56	0.19, 1.57	
Insurance			0.4			0.3			0.071
Commercial	—	—		—	—		—	—	
Medicare	0.68	0.40, 1.15		0.94	0.52, 1.68		1.44	0.89, 2.34	
Medicaid	1.13	0.62, 2.11		1.10	0.59, 2.10		0.80	0.48, 1.34	
Medicare/Medicaid	0.89	0.49, 1.66		1.50	0.75, 3.01		1.55	0.90, 2.70	
Veterans/public health service	1.05	0.42, 2.99		1.84	0.70, 4.91		1.87	0.82, 4.41	
Not insured	0.71	0.21, 2.54		1.19	0.34, 4.54		0.49	0.16, 1.37	
Unknown	5.57	0.61, 149		0.22	0.04, 1.12		0.28	0.05, 1.24	
RUCA Category			0.4			>0.9			>0.9
Urban	—	—		—	—		—	—	
Large rural	1.32	0.83, 2.13		1.08	0.65, 1.80		1.00	0.66, 1.52	
Small rural	0.80	0.46, 1.42		1.05	0.56, 1.96		0.95	0.57, 1.60	
Isolated rural	0.92	0.58, 1.46		0.97	0.59, 1.60		1.06	0.70, 1.61	
ADI Quintile			0.6			0.8			0.13
ADI_Q1	—	—		—	—		—	—	
ADI_Q2	0.98	0.58, 1.67		1.23	0.68, 2.21		0.98	0.60, 1.60	
ADI_Q3	1.25	0.72, 2.17		1.43	0.77, 2.64		1.39	0.84, 2.32	
ADI_Q4	1.38	0.81, 2.34		1.07	0.59, 1.91		0.86	0.53, 1.41	
ADI_Q5	1.37	0.72, 2.61		1.20	0.58, 2.47		0.78	0.43, 1.42	
Year of diagnosis			**0.002**			0.11			0.4
2017	—	—		—	—		—	—	
2018	1.11	0.63, 1.97		1.19	0.68, 2.11		1.39	0.86, 2.23	
2019	0.64	0.38, 1.08		1.56	0.90, 2.72		1.62	1.02, 2.58	
2020	0.55	0.33, 0.90		1.53	0.89, 2.65		1.25	0.80, 1.97	
2021	0.45	0.27, 0.74		0.84	0.49, 1.45		1.27	0.81, 2.00	
Stage at diagnosis			**<0.001**			**<0.001**			**<0.001**
Localized	—	—		—	—		—	—	
Regional	0.64	0.44, 0.91		31.2	20.2, 50.1		10.1	7.20, 14.3	
Distant	0.05	0.03, 0.08		11.6	7.19, 19.3		1.37	0.94, 1.99	

Abbreviations: CI = Confidence Interval, OR = Odds Ratio.
